# Detailed molecular and clinical investigation of a child with a partial deletion of chromosome 11 (Jacobsen syndrome)

**DOI:** 10.1186/1755-8166-2-26

**Published:** 2009-12-09

**Authors:** Emmanouil Manolakos, Sandro Orru, Rosita Neroutsou, Konstantinos Kefalas, Eirini Louizou, Ioannis Papoulidis, Loretta Thomaidis, Panagiotis Peitsidis, Sotirios Sotiriou, George Kitsos, Panagiota Tsoplou, Michael B Petersen, Aikaterini Metaxotou

**Affiliations:** 1 Bioiatriki S.A., Laboratory of Genetics, Athens, Greece; 2 Department of Medical Genetics, University of Cagliari, Binaghi Hospital, Cagliari, Italy; 3 Eurogenetica S.A., Thessaloniki, Greece; 4 Department of Pediatrics, University of Athens, Aglaia Kyriakou Children's Hospital, Athens, Greece; 5 Department of Fetal Medicine, Royal Free Hospital, UK; 6 Department of Embryology, University of Thessaly, Larissa, Greece; 7 Department of Ophthalmology, University of Ioannina, Ioannina, Greece; 8 Department of Genetics, Institute of Child Health, Athens, Greece

## Abstract

**Background:**

Jacobsen syndrome (JBS) is a rare chromosomal disorder leading to multiple physical and mental impairment. This syndrome is caused by a partial deletion of chromosome 11, especially subband 11q24.1 has been proven to be involved. Clinical cases may easily escape diagnosis, however pancytopenia or thrombocytopenia may be indicative for JBS.

**Results:**

We report a 7.5 years old boy presenting with speech development delay, hearing impairment and abnormal platelet function. High resolution SNP oligonucleotide microarray analysis revealed a terminal deletion of 11.4 Mb in size, in the area 11q24.1-11qter. This specific deletion encompasses around 170 genes. Other molecular techniques such as fluorescence in situ hybridization and multiplex ligation-dependent probe amplification were used to confirm the array-result.

**Discussion:**

Our results suggest that the identification and detailed analysis of similar patients with abnormal platelet function and otherwise mild clinical features will contribute to identification of more patients with 11q deletion and JBS.

## Background

Jacobsen syndrome (JBS) is a rare inherited disorder with variable phenotypic expression and partial deletion of chromosome 11q. To date more than 200 cases were reported, with an estimated prevalence of 1/100,000 births [1]. Clinical manifestations of JBS typically include developmental and mental retardation, facial dysmorphism, congenital heart defects, and thrombocytopenia [2-5]. In more detail typical JBS features include short stature, mental retardation, congenital heart defects, thrombocytopenia and characteristic facial dysmorphism consisting of skull deformities, ocular hypertelorism, ptosis, downward slanting palpebral fissures, epicanthal folds, flat nasal bridge, short nose with flat philtrum and thin upper lip, v-shaped mouth and small and low set ears. The neck is short, the hands show cutaneous syndactyly, the fingers are thin with flat finger pads and the feet are stubby, flat with clinodactylous toes. Malformations of kidneys are present in 13% of cases, gastrointestinal tract problems in 18%, abnormal genitalia in 36%, central nervous system and skeletal dysplasias in 14%. Abnormal platelet function, thrombocytopenia or pancytopenia is affecting at least 88% of cases and is usually present from birth [6,7]. About 20% of the children die during the first two years of life most commonly related to complications from congenital heart disease and less commonly from bleeding. Mental retardation is observed in 97% of cases while normal or borderline cognitive function is observed in less than 3% of cases. Behavioural problems such as attention deficit/hyperactivity disorders and psychiatric disorders have been rarely reported. Hearing impairment is not a common symptom but should be excluded in all patients with JBS. Immunological and hormonal problems may also be present. There is a wide range of severity of the clinical phenotype of JBS patients.

Dependent on the size of the 11q deletion, which usually lies between 7 and 20 Mb [8,9], the clinical features may vary also. Previous cytogenetic studies in JBS patients characterized the crucial band for the 11q monosomy syndrome as being 11q24.1 [10]. A genotype-phenotype correlation of 14 JBS patients found that 9 patients with a deletion of at least 12.1 Mb had severe global cognitive impairment, whereas the other 5 patients with deletion equal to or smaller than 11.8 Mb demonstrated mild cognitive impairment [11]. Partial expression of the JBS phenotype was observed in cases of very small terminal deletions or interstitial deletions within the JBS region [4,12,13].

Overall, many of the JBS symptoms are relatively unspecific. Especially in such cases with mild clinical symptoms, pancytopenia or thrombocytopenia may be suggestive for a JBS diagnosis [12,13].

Here we report a JBS case with abnormal platelet function, normal physical and mental development, mild facial dysmorphism, not age-appropriate language skills, and hearing impairment with partial deletion of distal chromosome 11q.

## Case presentation

The patient, a 7 years and 6 months old boy, was the second child of unrelated healthy parents. He was born by cesarean section after a full term pregnancy. His birth weight was 3,250 kg (50 percentile), length 50 cm (50 percentile) and head circumference (HC) 35.5 cm (50 percentile). His perinatal period was without problems. The motor development was normal as he sat independently at the age of 7 months and walked unassisted at the age of 11 months. His language development was delayed; first words were spoken later than aged 2 years and 6 months, and at 5 years he was referred to logotherapy. His parents described him as a healthy, sociable and smart boy with low linguistic skills. When the patient attended 1st grade in the mainstream school he was referred by his schoolteacher for full developmental assessment because of learning difficulties and speech developmental delay.

On physical examination he was quite a sociable child, with mild dysmorphic facial features such as ocular hypertelorism, downward slanting palpebral fissures, epicanthal folds, flat nasal bridge, short nose with flat philtrum, and thin upper lip. Additionally, his neck was short and the feet were stubby and flat. His weight was 27 kg (50th percentile), height 122 cm (25th percentile) and his HC 52 cm (25th percentile). On developmental examination: his nonverbal skills were equivalent to a 5 years 9 months level with good language understanding, being able to spell, read and write small words, but his oral speech development was very poor. His expressive language was limited to small words and phrases with many phonological difficulties. According to Griffiths Mental Development Scales and Bayley Scales of Infant Development (2nd edition) his General Developmental Quotient (GDQ) was 78 with performance DQ of 86 and language DQ of 61. His behavior was normal for developmental age.

On neurological examination he was slightly hypotonic but without asymmetry. Heart auscultation revealed a mild systolic murmur. Audiological examination showed a mild bilateral selective high frequency hearing loss (4000-8000 Hz). Heart ultrasound (triplex) showed a single papillary muscle resulting in mild mitral valve regurgitation but without hemodynamic changes. The echocardiogram was normal. Full ophthalmologic examination was normal. Hematological tests revealed anemia with Hb of 9.3 g/100 ml, Ht of 30%, MCV of 57.9, MCH of 18, and MCHC of 31 g/100 ml, with normal blood cell and platelet counts 269,000/μl (140,000/μl-300,000/μl). Bleeding time was very elongated >17 min (2-4 min) and platelet aggregation test (PSA) was abnormal 196 sec (<142 sec).

Results of further biochemical tests were the following: urea: 34 mg/dl, blood sugar: 86 mg/dl, creatinine: 0.35 mg/dl, calcium: 9.4 mg/dl, Mg: 2.5 mg/dl, K: 4.8 mEq/l, Na: 140 mEq/l, Cl:106 mEq/l, SGOT: 40 U/l, SGPT: 30 U/l, GT: 8 U/l, cholesterol: 132 mg/dl, HD: 61 mg/dl, LDL: 59 mg/dl, CPK: 65 u/l, and ammonia: 0.69 μg/dl. Thyroid function and kidney-liver-spleen ultrasound were also normal.

## Results

**Figure 1:**
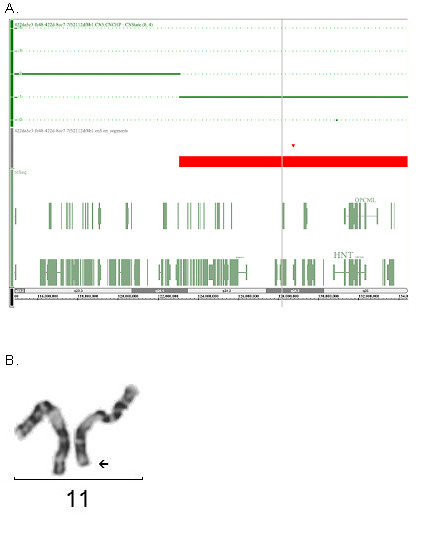
**A. SNP Oligonucleotide Microarray Analysis (SOMA) identifies the deletion in the long arm of chromosome 11**. The deleted segment on chromosome 11 lies between marker SNP_A-2122040 at chr11: 123073531 and marker SNP_A-2246844 at chr11: 134449982, spanning an area of 11.37 Mb. **B. Partial karyotype of chromosomes 11 of the proband**. Arrow shows the deleted chromosome segment.

Chromosome analysis of the patient was performed using GTG-banding techniques on stimulated blood lymphocytes. Cytogenetics revealed a deletion of the long arm of one chromosome 11 in the band q24.1 (Fig. 1B). Fluorescence *in situ *hybridization (FISH) using a commercially available subtelomeric probe (Abbott/Vysis) for 11qter confirmed the suggested deletion. The parental karyotypes were normal, suggesting a de novo appearance of the deletion.

The multiplex ligation-dependent probe amplification (MLPA) technique was performed using the SALSA P036B probe set (MRC-Holland) containing probes for all subtelomeric regions, which confirmed the deletion (data not shown). Finally, single nucleotide polymorphism (SNP) oligonucleotide microarray analysis (SOMA) was performed on DNA of the patient. Thus, the size of the deletion was determined as 11.4 Mb, in the region 11q24.1-11qter. The deleted segment extended from 123,073,531 to 134,449,982 bps on chromosome 11 (Fig. 1A).

## Discussion

We report a 7.5 years old boy with an 11.4 Mb terminal deletion of 11q24.1-qter characterized by high-resolution SNP oligonucleotide microarray. The detected size of the deletion and the observed comparatively mild symptoms fit well to the previously defined cut off size of 11.8 Mb for mild cases [12]

Tyson et al. [14] suggest that a critical region for the conotruncal heart defect associated with JBS may lie within a region spanning between 129.03 and 130.6 Mb, which contains *ADAMTTS8*, a gene involved in angiogenesis [15]. Polymorphisms in the *SNX19 *gene have been proposed to be associated with coronary disease [16] and the *JAM3 *gene has previously been proposed as a candidate gene for the JBS cardiac phenotype. The megakaryocytic defects in 14 Jacobsen syndrome patients were mapped to a minimal region of overlap in 11q that includes the *FLI1 *gene, thus suggesting that dysmegakaryopoiesis in these patients may be caused by hemizygous loss of *FLI1 *[17]. The heart defect present in our case was a mitral valve regurgitation, which is not a heart defect classically described in JBS.

Thrombocytopenia in JBS is usually chronic. Abnormal platelets are detected with giant granules and the bone marrow shows many micromegakaryocytes. Moreover, electron microscopy reveals granule fusion within blood platelets [6]. In our patient hemizygosity of the FLI1 gene due to deletion of 11q24.3 was present together with abnormal platelet function, which had passed notice up to 7 years of age.

Like in our case, it is important to keep in mind that the identification and further delineation with array-CGH techniques of similar patients with mild features will contribute to understand the genetic spectrum of the 11q phenotype.

## Materials and methods

### SNP Oligonucleotide Microarray Analysis (SOMA)

SOMA was performed using the Affymetrix Genome Wide Human SNP Array 6.0, which includes over 906,600 single nucleotide polymorphisms (SNPs) and more than 946,000 probes for the detection of copy number variation (CNV) in the human genome. Sample preparation, hybridization and scanning were performed using GeneChip^® ^Instrument System hardware according to the manufacturer's specifications (Affymetrix, Santa Clara, CA). Analysis was performed using the Affymetrix Genotyping Console software (version 3.0.1). The samples met Affymetrix recommended values for Contrast Quality Control (QC) (SNP) and Median of the Absolute Values of all Pairwise Differences (MAPD) QC (CNV). Data from both SNP and copy number probes were used to identify copy number aberrations compared to an internal reference set. The segment report was restricted to regions of 100 kb or greater with 10 or more consecutive probes that differed significantly from the expected normalized diploid values. The map positions of the deleted segment refer to the Genome Assembly May 2004 (Build 35).

### MLPA Analysis

MLPA with SALSA P036B and P070 probe mixes (MRC-Holland) was performed in a GeneAmp PCR System 2700 (Applied Biosystems, Foster City, CA, USA) as recommended by the manufacturer's protocol. PCR products were electrophoresed in an ABI Prism 3100 Genetic Analyser and analysed with the GeneMapper 3.5 software package (Applied Biosystems). Sequence deletion or duplication was considered when a 35-50% variation of the relative peak area of the amplification product of the respective probe was obtained. If no concordant results between the two probe mixes were obtained, the assay was repeated.

### Cytogenetics and FISH Analysis

Chromosome analysis was performed using GTG-banding techniques on stimulated blood lymphocytes and analyzed at 550-600 band resolution. Fluorescence in situ hybridization (FISH) studies were performed using a set of probes specific for the subtelomere of the 11q chromosome (Vysis/Abbott). Metaphase chromosomes were obtained and fixed cell suspensions were dropped onto clean microscope slides. A measure of 2.5 μl of the probe was then placed on the glass slide with a coverslip. Coverslips were sealed with rubber cement and slides were placed in a moist chamber and incubated overnight at 37°C. The slides were washed and counterstained with DAPI, and cells were examined with a Zeiss Axioplan II, Imager.M1 or Imager.Z1 fluorescence microscope equipped with a triple-bandpass filter. Digital images were captured and stored with Isis software V 3.4.0 (Metasystems, Altlussheim, Germany).

## Consent

Written informed consent was obtained from the parents of this patient for publication of this case report. A copy of the written consent is available for review by the Editor-in-Chief of this journal.

## Competing interests

The authors declare that they have no competing interests.

## Authors' contributions

EM wrote the manuscript; SS and LT referred the patient for study; LT and PP coordinated the clinical analysis of the patient; EM and RN performed the cytogenetic analysis; PT, KK and LE signed out the molecular cytogenetic results; SO, IP were responsible for the MLPA analysis; GK performed the ophthalmologic examination; MBP and MA coordinated the study. All authors have read and approved the manuscript.
